# The association between patellar tendon stiffness measured with shear-wave elastography and patellar tendinopathy—a case-control study

**DOI:** 10.1007/s00330-020-06952-0

**Published:** 2020-06-04

**Authors:** Stephan J. Breda, Arco van der Vlist, Robert-Jan de Vos, Gabriel P. Krestin, Edwin H. G. Oei

**Affiliations:** 1 Department of Radiology & Nuclear Medicine, Erasmus MC University Medical Center, Doctor Molewaterplein 40, 3015 GD Rotterdam, The Netherlands; 2 Department of Orthopedics & Sports Medicine, Erasmus MC University Medical Center, Doctor Molewaterplein 40, 3015 GD Rotterdam, The Netherlands

**Keywords:** Patellar ligament, Elasticity imaging techniques, Tendinopathy, Athletes, Reproducibility of results

## Abstract

**Objectives:**

(1) To determine the association between patellar tendon stiffness and the presence of patellar tendinopathy (PT). (2) To evaluate the reliability of shear-wave elastography (SWE).

**Methods:**

Participants were consecutively enrolled between January 2017 and June 2019. PT was diagnosed clinically and confirmed by either grayscale US or power Doppler US, or both. Controls had no history of anterior knee pain and no clinical signs of PT. Patellar tendon stiffness (kilopascal, kPa) was assessed using SWE. Logistic regression was used to estimate adjusted odds ratios (ORs) and 95% confidence intervals (CIs). Reliability analyses included coefficients-of-variation (CV), coefficients-of-repeatability (CR), intraclass correlation coefficient (ICC) for intraobserver and interobserver reliability, and Bland-Altman analysis.

**Results:**

In total, 76 participants with PT (58 men, mean age 24.4 ± 3.8 years) and 35 asymptomatic controls (16 men, mean age 21.5 ± 3.8 years) were included. Univariate analyses (OR 1.094, 95% CI 1.061–1.128, *p* < .001) and adjusted multivariate analyses (OR 1.294, 95% CI 1.044–1.605, *p* = .018) showed that athletes with PT had significantly increased patellar tendon stiffness. ICC for intraobserver reliability was 0.95 (95% CI 0.92–0.97), CR (CV) 12 kPa (10%) and 0.79 (95% CI 0.65–0.88), CR (CV) 18 kPa (21%) for interobserver reliability. Mean differences from Bland-Altman analysis were 5.6 kPa (95% CI 3.1–8.1, *p* < .001) for intraobserver reliability and 4.6 kPa (95% CI 1.9–7.2, *p* < .001) for interobserver reliability.

**Conclusions:**

PT is associated with significantly higher patellar tendon stiffness. SWE measurements demonstrate excellent intraobserver reliability and good interobserver reliability. Therefore, SWE is a promising tool to implement in longitudinal studies and future studies should evaluate its prognostic value and utility as a monitoring tool in athletes with PT.

**Key Points:**

*• Patellar tendon stiffness measured with shear-wave elastography (SWE) is higher in athletes with patellar tendinopathy than in healthy controls, also after adjusting for potential confounders.*

*• Excellent intraobserver reliability and good interobserver reliability were found for the quantitative assessment of patellar tendon stiffness using SWE.*

## Introduction

Patellar tendinopathy (PT) is an overuse injury of the patellar tendon resulting in pain, decreased exercise tolerance, and impaired function [1]. PT is highly prevalent in jumping athletes, with reported rates of 45% for elite volleyball players and 32% for elite basketball players [2]. There is consensus that PT is a clinical diagnosis with focal load-related pain, established by medical history taking and clinical examination. Currently, the applicability of ultrasound (US) is limited to confirming the clinical diagnosis of PT by assessing morphological changes [3]. Tendinopathy-related abnormalities on US are tendon thickening with hypoechoic areas and/or increased Doppler flow [4, 5]. These alterations are associated with tendinopathy; however, they have also been reported in up to 59% of asymptomatic athletes [6]. Therefore, changes in tendon structure on grayscale US (GSUS) are considered a risk factor for tendinopathy rather than indicative for PT or tendon pain [7]. Alternative imaging techniques that better reflect pain remain to be investigated as they could provide attractive novel biomarkers to assess therapy response.

Shear-wave elastography (SWE) is an ultrasound-based imaging technique which evaluates viscoelastic properties, depicted as color-coded images (elastogram) [8]. Accordingly, SWE offers additional information to structural changes observed with GSUS. SWE assesses tendon stiffness both qualitatively and quantitatively by acquiring velocity measurements of directional propagating shear-waves generated by focused ultrasound pulses [9]. The assessment of patellar tendon stiffness using SWE could potentially correlate better with experienced pain in athletes with PT. Moreover, the superficial location of the patellar tendon facilitates implementation of SWE.

Musculoskeletal applications of SWE constitute a relatively new area which has emerged from well-established applications in breast, liver, thyroid, and prostate imaging [10–13]. Additionally, SWE has already shown potential to discriminate between athletes with unilateral PT and asymptomatic athletes [14]. However, recent studies reported conflicting SWE outcomes in PT [15]. Consequently, the association between patellar tendon stiffness measured with SWE and the presence of PT and the reliability of SWE are still largely unknown.

The primary aim of this study was, therefore, to determine the association between patellar tendon stiffness and the presence of PT in jumping athletes. The secondary aim was to evaluate the reliability of the patellar tendon stiffness assessment and image analysis using SWE.

## Materials and methods

This case-control study in Erasmus MC University Medical Center Rotterdam, The Netherlands, was approved by the institutional review board. Participants provided written informed consent prior to inclusion. We performed cross-sectional analysis of baseline data from a prospective trial investigating two different exercise programs for PT (ClinicalTrials.gov, ID: NCT02938143).

### Study participants

Participants were consecutively enrolled. National sports federations and regional healthcare providers facilitated recruitment. Athletes performing sports involving frequent jumping or cutting maneuvers were eligible. Potential subjects underwent initial online screening to assess the location of tenderness on a self-reported pain map [16]. The Victorian Institute of Sports Assessment questionnaire for patellar tendons (VISA-P) was administered to measure symptoms, function, and ability to play sports [17]. A VISA-P < 80 was one of the inclusion criteria for PT [18]. All eligibility criteria are listed in Table 1.

**Table 1:** Inclusion and exclusion criteria

	Inclusion criteria	Exclusion criteria
Asymptomatic athletes	Age 18–35 years	Acute knee or patellar tendon injuries
	Playing patellar tendon-loading sports for at least 3 times a week	Prior knee surgery without full rehabilitation
	No history of anterior knee pain or diagnosis of PT	Known presence of inflammatory joint diseases or familial hypercholesterolemia
	VISA-P score 100/100 points	Daily use of drugs with a putative effect on the patellar tendon in the preceding 12 months (e.g., fluoroquinolones)
		Local injection therapy with corticosteroids in the preceding 12 months
		Previous patellar tendon rupture
Patellar tendinopathy	Age 18–35 years	Acute knee or patellar tendon injuries
	Playing patellar tendon-loading sports for at least 3 times a week	Prior knee surgery without full rehabilitation
	History of anterior knee pain located in the trajectory of the patellar endon or its patellar or tibial insertion in association with training and competition	Known presence of inflammatory joint diseases or familial hypercholesterolemia
	Tenderness on palpation in the corresponding painful area	Daily use of drugs with a putative effect on the patellar tendon in the preceding 12 months (e.g., fluoroquinolones)
	Symptom duration of at least 2 weeks	Local injection therapy with corticosteroids in the preceding 12 months
	VISA-P score < 80/100 points	Previous patellar tendon rupture
	On ultrasound, presence of structural and/or hypoechoic changes of highly organized fiber bundles and/or tendon thickening (anterior-posterior diameter > 6 mm) and/or the presence of Doppler flow detected with PDUS.	Daily exercise therapy with a minimum duration of 4 weeks in total in the preceding 12 months
		Contraindications for MRI

*PT*, patellar tendinopathy; *VISA-P*, Victorian Institute of Sports Assessment questionnaire for patellar tendons; *PDUS*, power Doppler ultrasound

### Inclusion protocol

Jumping athletes with suspected PT and asymptomatic athletes were invited to our hospital to confirm eligibility. Clinical evaluation was performed by a sports physician (R.V.) with 10 years’ experience, and athletes were regarded positive for PT if tenderness at the inferior patellar pole or patellar tendon could be reproduced on palpation and a single-leg squat [19]. Provocation tests of the patellofemoral joint were performed to exclude patellofemoral pain [20]. Subsequently, GSUS and power Doppler US (PDUS) were performed to verify the clinical diagnosis. US criteria for PT were presence of structural and/or hypoechoic changes and/or tendon thickening (anterior-posterior diameter > 6 mm) and/or the presence of intratendinous Doppler flow [21]. We defined our reference standard for having PT as a clinical diagnosis with affirmative findings on GSUS and/or PDUS. For athletes with bilateral PT, the individual selected the most painful side. Asymptomatic athletes who had a maximum VISA-P score (100/100) and no history of anterior knee pain or diagnosis of PT were used as controls (Table 1). GSUS and PDUS were acquired, but findings were not an eligibility criterion in this group. Weight and height measures were used to calculate body mass index (kg/m^2^). Activity level was assessed using the Cincinnati Sports Activity Scale (CSAS) [22].

### Imaging methods

US was performed by one trained examiner (S.B.: radiologist-in-training with 5 years’ experience) using an ultrasound machine equipped with SWE (LOGIQ E9, GE Healthcare). A linear 5–15-MHz transducer (ML6-15, GE Healthcare) was used for GSUS and PDUS and a linear 3.1–10-MHz transducer (9L, GE Healthcare) for SWE. Ultrasound gel (Sonogel Vertriebs GmbH) was used at room temperature (21 °C).

Participants were examined in supine position with the back rest of the examination table upright in 60° for patient comfort and improved patellar tendon relaxation. GSUS was performed with both knees in 30° flexion, supported by a foam roll. PDUS and SWE were performed in passive extension of both knees. The standardized US acquisition protocol included longitudinal and transverse GSUS of the patellar tendon and transverse cine-loops for PDUS. The patellar tendon was designated as vascular if it demonstrated one or more blood vessels in the posterior portion of the patellar tendon or within the tendon. SWE was performed with mild pressure, in the longitudinal plane with the inferior patellar pole just in the field-of-view. Elastograms were generated in dual-screen mode, displaying GSUS and the overlaying elastogram. Three elastograms were acquired, of which one was randomly selected for the first analysis directly after the image acquisition. A second analysis of all elastograms in PT athletes was performed by the same examiner (S.B.) after the recruitment of subjects had finished, blinded for the results of the first analysis. The second analysis consisted of stiffness measurements in all three elastograms acquired using the same method as the first analysis. Patellar tendon stiffness was averaged for the three elastograms and the relative variability of these measurements was calculated. The maximum thickness of subcutaneous tissue overlying the proximal patellar tendon was measured on a transverse GSUS image, at a standardized location within 1 cm below the inferior patellar border. A subset of controls was invited consecutively to be re-examined with SWE at the same time point by an independent examiner (A.V.) with 2 years’ experience, who also performed the analyses of these collected images, blinded for the results of the first examiner. Quantitative analysis of patellar tendon stiffness was performed on the ultrasound machine, with maximum transparency of the elastograms to avoid subjective placements of regions-of-interests (ROIs). A reference ruler of 20 mm was set posterior to the patellar tendon, starting 5 mm distal to the inferior patellar pole. This guided placement of circular ROIs and avoided inclusion of artifacts from the patella (Fig. 2). ROIs were not fixed in size or number. Median tendon stiffness (kPa) was calculated for each ROI and overall median stiffness including all ROIs. The separate ROIs were labeled “ROI1-ROI4” from proximal to distal in the proximal patellar tendon.

### Statistical analysis

SPSS software (version 25; IBM Corp.) was used. Normal distribution was tested using Shapiro-Wilk’s test. Median and interquartile range (IQR) were obtained for non-normally distributed data. Between-group differences were assessed with Student’s *t* test for normally distributed data and Mann-Whitney *U* test for non-normally distributed data. Analyses included the influence of tendon calcifications on patellar tendon stiffness in specific ROIs. In athletes with unilateral PT, we compared patellar tendon stiffness between the symptomatic and the asymptomatic patellar tendon. Logistic regression analysis was performed to calculate odds ratios (ORs) and 95% confidence intervals (95% CIs). Univariate (unadjusted) and multivariate models adjusted for potential confounders, including age, sex, body mass index, and thickness of subcutaneous tissue, were applied. Determinants with *p* value < .10 were used in the multivariable model. Interaction terms for age*stiffness and sex*stiffness were added, based on findings in previous research [23, 24]. Multicollinearity was tested using variance inflation factor (VIF), with an acceptable maximum of 2.5. The relative variability of the three SWE measurements was assessed using coefficient-of-variation (CV) and coefficient-of-repeatability (CR). The intraobserver reliability for the analyses of the different elastograms and interobserver reliability for independent SWE acquisitions and analyses were assessed using CV, CR, intraclass correlation coefficient (ICC), and Bland-Altman analysis [25–27]. An ICC value reflected “poor” (less than 0.5), “moderate” (between 0.5 and 0.75), “good” (between 0.75 and 0.9), and “excellent” (greater than 0.90) [28]. *P* values < .05 were considered statistically significant.

## Results

### Study population

**Fig. 1:**
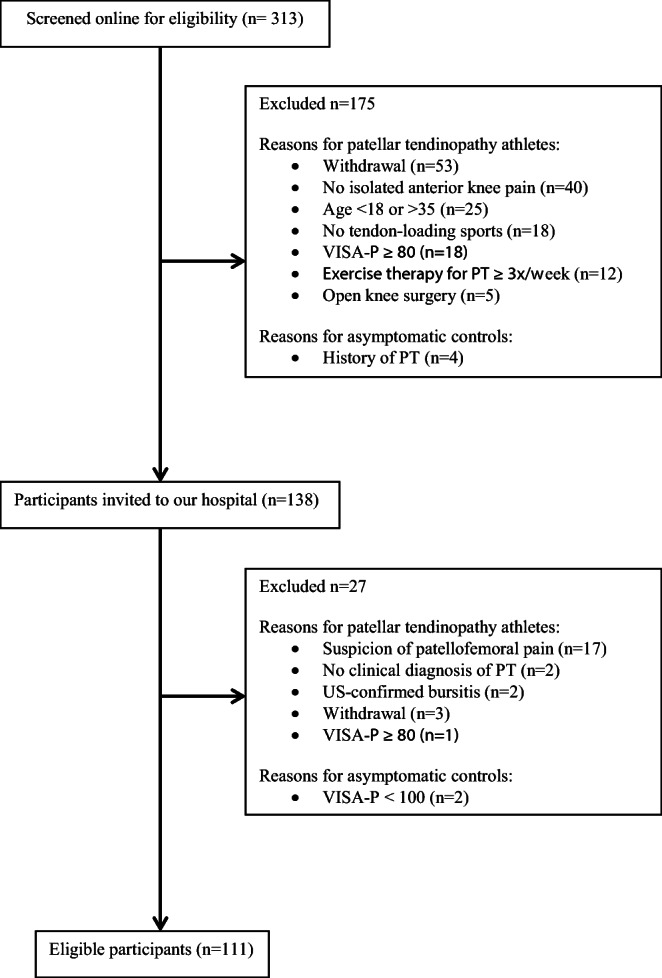
Recruitment flowchart of PT athletes and asymptomatic controls

Participants were consecutively enrolled between January 2017 and June 2019. A total of 313 applications from potentially eligible PT athletes and asymptomatic controls were initially screened, of which 138 participants were invited to our hospital to verify or exclude the diagnosis of PT. Finally, 111 participants remained eligible for inclusion (Fig. 1). Clinical and demographic characteristics of the study population are listed in Table 2. Participants with PT were significantly older, had higher BMI, and consisted of more men than asymptomatic controls. Athletes with PT (*n* = 76) participated in volleyball (*n = 26*), soccer (*n = 17*), basketball (*n = 16*), korfball (*n = 8*), track and field (*n = 4*), field hockey (*n = 3*), and handball (*n = 2*) as primary sports. Asymptomatic controls (*n* = 35) participated in basketball (*n = 15*), korfball (*n = 10*), volleyball (*n = 9*), and track and field (*n = 1*). No significant differences were found in activity levels between athletes with PT and asymptomatic controls. In PT athletes, the left patellar tendon was the primary site of symptoms in 41 participants (54%) and the right patellar tendon in 35 participants (46%). The diagnosis of PT was unilateral in 44 participants (58%), of which 26 were left-sided. Median duration of symptoms in PT athletes was 104 weeks (IQR, 43–208 weeks).

**Table 2:** Baseline characteristics of participants

Characteristic	Asymptomatic athletes (*n* = 35)	Patellar tendinopathy (*n* = 76)	*p* value
Mean age (year)	21.4 ± 3.8	24.4 ± 3.8	< .001
No. of men	18 (51)	58 (76)	.003
Mean height (cm)	180.1 ± 10.3	184.7 ± 9.3	.02
Mean weight (kg)	71.0 ± 9.5	81.8 ± 12.3	< .001
Mean BMI (kg/m^2^)	21.9 ± 1.8	23.9 ± 2.9	< .001
Mean clinical score (VISA-P)	100 ± 0	55 ± 13	< .001
Sports activity scale (CSAS)			.10
Level I (4 to 7 days/week)			
100	8 (23)	17 (22)	
95	0 (0)	0 (0)	
90	0 (0)	0 (0)	
Level II (1 to 3 days/week)			
85	27 (77)	50 (66)	
80	0 (0)	9 (12)	

Data are means ± standard deviation except where they are numbers of participants and data in parentheses are percentages. *BMI*, body mass index; *VISA-P*, Victorian Institute of Sports Assessment questionnaire for patellar tendons; *CSAS*, Cincinnati Sports Activity Scale

### GSUS and PDUS findings

The proximal patellar tendon was significantly thicker in PT athletes (mean 8.4 ± 2.4 mm) than in asymptomatic controls (mean 4.1 ± 0.9 mm) (*p* < .001). Hypoechoic changes were seen in 89% of PT athletes and 26% of asymptomatic controls. Tendon calcifications were observed in 27% of PT athletes and erosions of the inferior patellar border in 29%. Both were absent in asymptomatic controls. Intratendinous Doppler flow was present in 89% of PT athletes and 3% of asymptomatic controls.

### SWE findings

**Fig. 2:**
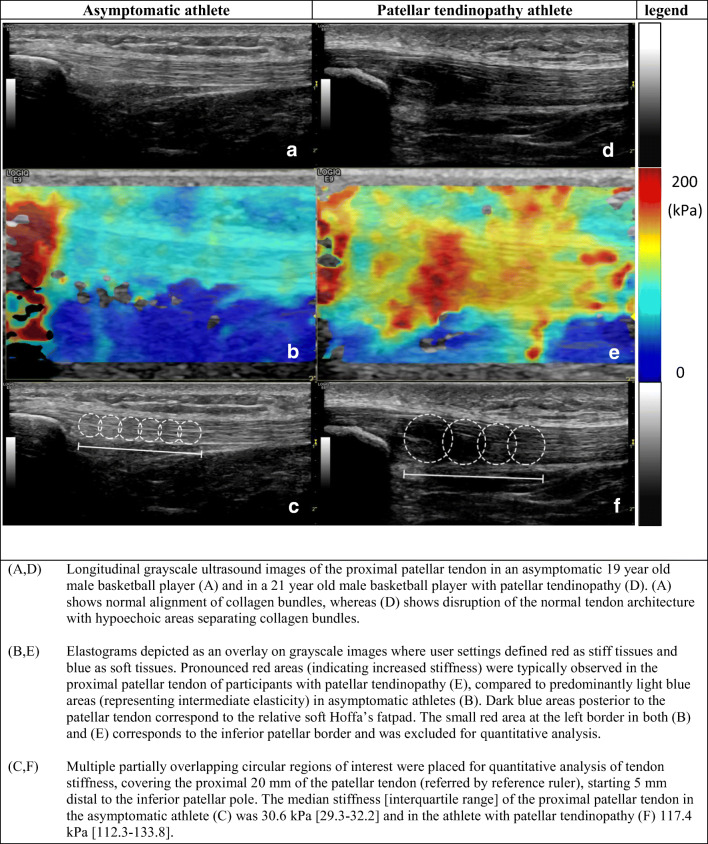
Grayscale US and corresponding shear-wave elastograms in an asymptomatic athlete and an athlete with patellar tendinopathy. **a**, **d** Longitudinal grayscale ultrasound images of the proximal patellar tendon in an asymptomatic 19-year-old male basketball player (**a**) and in a 21-year-old male basketball player with patellar tendinopathy (**d**). **a** shows normal alignment of collagen bundles, whereas **d** shows disruption of the normal tendon architecture with hypoechoic areas separating collagen bundles. **b**, **e** Elastograms depicted as an overlay on grayscale images where user settings defined red as stiff tissues and blue as soft tissues. Pronounced red areas (indicating increased stiffness) were typically observed in the proximal patellar tendon of participants with patellar tendinopathy (**e**), compared to predominantly light blue areas (representing intermediate elasticity) in asymptomatic athletes (**b**). Dark blue areas posterior to the patellar tendon correspond to the relative soft Hoffa’s fatpad. The small red area at the left border in both **b** and **e** corresponds to the inferior patellar border and was excluded for quantitative analysis. **c**, **f** Multiple partially overlapping circular regions of interest were placed for quantitative analysis of tendon stiffness, covering the proximal 20 mm of the patellar tendon (referred by reference ruler), starting 5 mm distal to the inferior patellar pole. The median stiffness (interquartile range) of the proximal patellar tendon in the asymptomatic athlete (**c**) was 30.6 kPa (29.3–32.2) and in the athlete with patellar tendinopathy (**f**) 117.4 kPa (112.3–133.8)

Stiffness of the proximal patellar tendon was significantly higher in PT athletes (median 74.9 kPa, IQR [56.4–105.4]) than in asymptomatic athletes (median 35.6 kPa, IQR [29.9–43.0]) (*p* < .001) (Fig. 2). In PT athletes, no significant difference in patellar tendon stiffness was found between primary left-symptomatic athletes and primary right-symptomatic athletes (*p =* .360). Only in ROI 1, patellar tendon stiffness was significantly higher in PT with tendon calcifications than in PT without calcifications (*p =* .017). In PT athletes without tendon calcifications, symptomatic tendons were still significantly stiffer than asymptomatic tendons in ROI 1, both on the left (*p =* .043) and right (*p =* .005) side, but not in other ROIs. This increased stiffness in ROI 1 was not observed in the asymptomatic tendons (left *p =* .679 and right *p* = .396).

### Variability of the SWE measurements in PT athletes

For the patellar tendon stiffness assessments in all ROIs, the CV was 5.3% (95% CI 4.0–6.3) and the CR was 6.6 kPa (IQR 3.6–12.1). For analysis in separate ROIs, the CV ranged from 10.8 to 11.8% and the CR ranged from 7.3 to 10.7 kPa.

### Association between patellar tendon stiffness and PT

Patellar tendinopathy was associated with significantly higher patellar tendon stiffness, both in univariate analyses (OR 1.094, 95% CI 1.061–1.128, *p* < .001) and in adjusted multivariate regression analyses (OR 1.294, 95% CI 1.044–1.605, *p* = .018). The odds ratios for patellar tendon stiffness are estimated for each kilopascal (kPa). In univariate analysis, 7 determinants were associated with the presence of PT symptoms with a *p* value < 0.10, and therefore included in the multivariate model (Table 3). The variance inflation factors were well within the acceptable limit (range VIF, 1.25–1.56).

**Table 3:** The association between patellar tendon stiffness and patellar tendinopathy

Determinant	Univariable	Multivariable^a^
Age at T0	*1.226 (1.114–1.350)*	1.407 (0.924–2.144)
Male sex	*3.412 (1.683–6.916)*	5.663 (0.090–355.276)
Index knee: left	1.111 (0.580–2.128)	
Body mass index at T0	*1.533 (1.268–1.853)*	*2.380 (1.554–3.642)*
Subcutaneous tissue (mm)	*0.545 (0.373–0.795)*	*0.365 (0.152–0.877)*
Cincinnati Sports Activity Scale	0.985 (0.937–1.035)	
Patellar tendon stiffness (kPa)	*1.094 (1.061–1.128)*	*1.294 (1.044–1.605)*^*b*^
Age*stiffness	*1.004 (1.003–1.006)*	0.994 (0.986–1.003)
Sex*stiffness	*1.034 (1.022–1.046)*	0.967 (0.890–1.049)

Data are presented as odds ratio (95% CI); data with *p* < .05 are italicized in univariable model

^a^Determinants with *p* < .10 by univariable logistic regression were used in the multivariable model

^b^Odds ratio for patellar tendon stiffness assessed with shear-wave elastography is estimated for each kilopascal (kPa)

### Intraobserver reproducibility of SWE

**Fig. 3:**
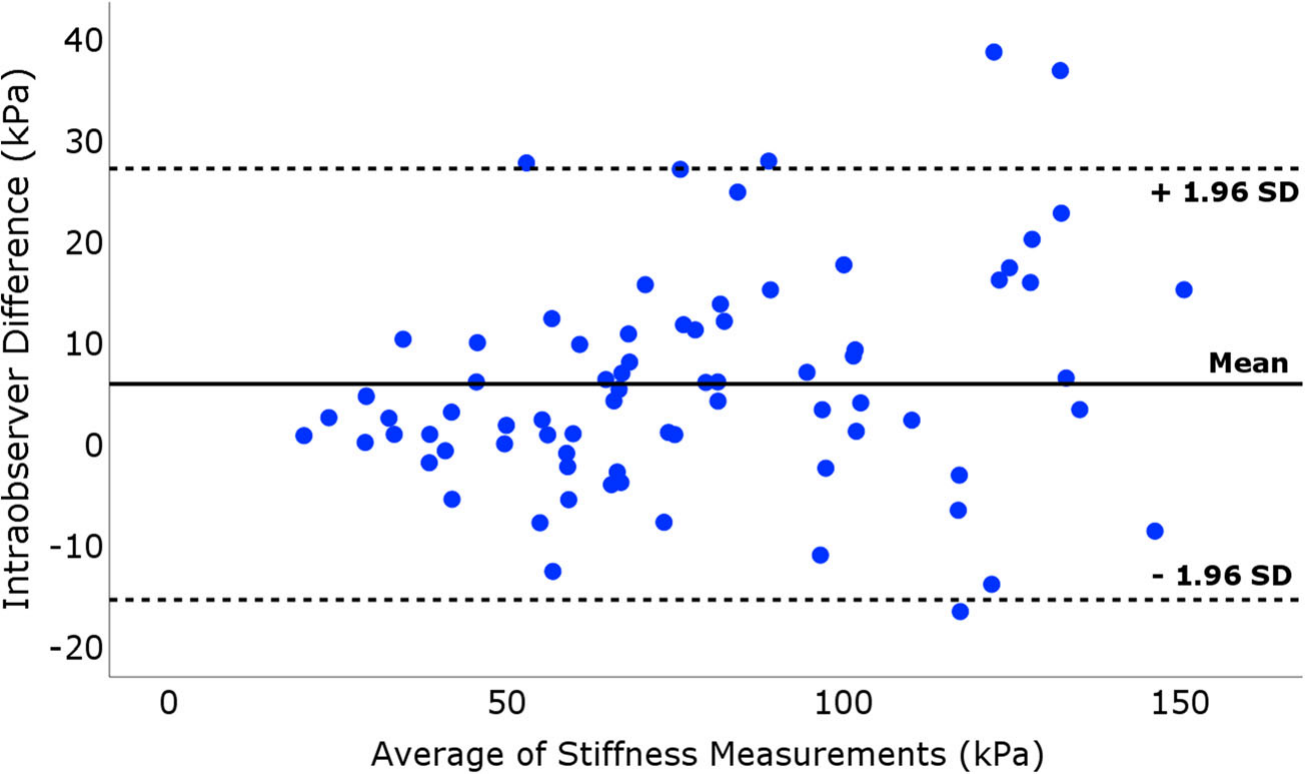
Intraobserver reliability of SWE in seventy-six athletes with patellar tendinopathy. Bland-Altman plot illustrating the intraobserver reliability for the patellar tendon stiffness assessment using SWE. The differences between each pair of the stiffness measurements plotted on the *y*-axis are shown against the mean of these measurements on the *x*-axis. The solid line represents the mean value and dashed lines represent the limits of agreement, defined as mean ± 1.96SD

The intraobserver reliability analysis (Table 4) revealed an intraclass correlation coefficient (ICC) of 0.95 (95% CI 0.92–0.97) for the median patellar tendon stiffness using all ROIs between analysis 1 (median stiffness 74.9 kPa [56.4–105.4]) and analysis 2 (median stiffness 69.9 kPa [54.7–100.3]). The coefficient-of-repeatability (CR) and coefficient-of-variation (CV) were 11.9 kPa [5.1–24.9] and 10.3% (95% CI 7.9–12.2), respectively. For the separate ROIs, the ICC ranged from 0.85 to 0.92 and CR (CV) from 13.3 to 20.2 kPa (15.1–19.2%). The mean difference from Bland-Altman analysis (Fig. 3) was 5.6 kPa (95% CI 3.1–8.1, *p* < .001) and limits of agreement were −15.8 kPa (lower limit) and 26.9 kPa (upper limit).

**Table 4:** Intraobserver reliability analysis of patellar tendon stiffness in seventy-six athletes with patellar tendinopathy (*N* = 76 tendons)

	Analysis 1 (SB)	Analysis 2 (SB)	Intraobserver reliability		
Location	Stiffness (kPa)	Stiffness (kPa)	CV (%)^a^	CR (kPa)^b^	ICC (95%CI)^c^
All ROIs	74.9 [56.4–105.4]	69.9 [54.7–100.3]	10.3 (7.9–12.2)	11.9 [5.1–24.9]	0.95 (0.92–0.97)
ROI 1	78.3 [51.6–117.3]	78.4 [51.7–111.6]	15.8 (9.2–20.3)	14.8 [5.3–26.8]	0.92 (0.88–0.95)
ROI 2	85.4 [55.9–127.6]	72.4 [54.4–111.8]	15.1 (12.3–17.4)	16.2 [7.0–44.4]	0.89 (0.83–0.93)
ROI 3	69.7 [52.7–102.3]	63.8 [49.2–84.5]	19.2 (13.3–23.6)	20.2 [6.8–41.6]	0.85 (0.77–0.90)
ROI 4	59.9 [41.7–76.3]	48.9 [34.1–61.9]	18.9 (11.2–24.3)	13.3 [5.5–24.3]	0.92 (0.85–0.95)

Patellar tendon stiffness was assessed using shear-wave elastography (SWE), expressed as median (interquartile range) in kPa

^a^CV: coefficient-of-variation (%), 95% confidence interval

^b^CR: coefficient-of-repeatability (kPa), also referred to as the smallest real difference (SRD)

^c^ICC: intraclass correlation coefficient (ICC), 95% confidence interval

### Interobserver reproducibility of SWE

**Fig. 4:**
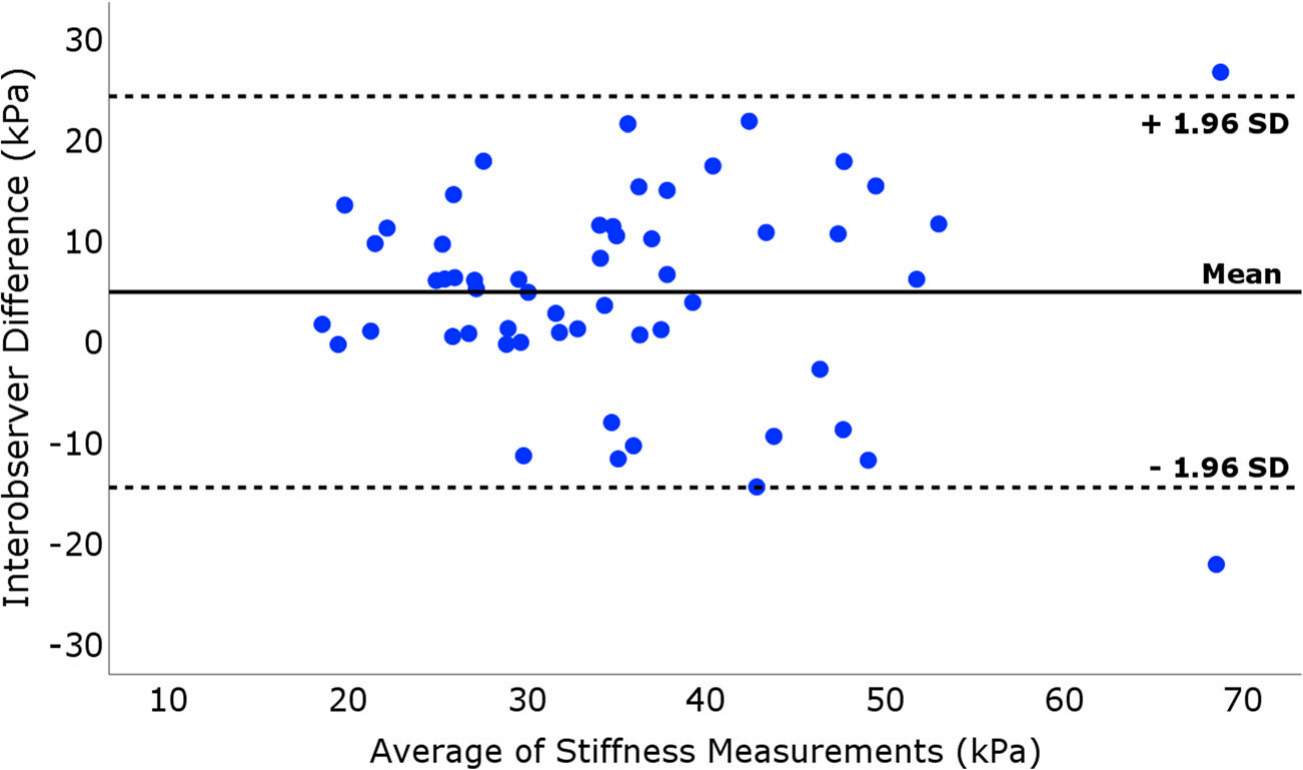
Interobserver reliability of bilateral SWE in twenty-eight healthy athletes. Bland-Altman plot illustrating the interobserver reliability for the patellar tendon stiffness assessment using SWE. The differences between each pair of the two examiners’ stiffness measurements plotted on the *y*-axis are shown against the mean of these measurements on the *x*-axis. The solid line represents the mean value and dashed lines represent the limits of agreement, defined as mean ± 1.96SD

For the interobserver reproducibility, 56 paired measurements in 28 healthy athletes were used (Table 5). The ICC between examiner 1 (S.B.) and examiner 2 (A.V.) was 0.79 (95% CI 0.65–0.88) and CR (CV) was 18 kPa (21%). The mean difference from Bland-Altman analysis (Fig. 4) was 4.6 kPa (95% CI 1.9–7.2, *p* < .001) and the limits of agreement were −14.8 kPa (lower limit) and 24.0 kPa (upper limit).

**Table 5:** Interobserver reliability analysis of patellar tendon stiffness in twenty-eight healthy athletes (*n* = 56 tendons)

	Examiner 1 (SB)	Examiner 2 (AV)	Interobserver reliability		
Location	Stiffness (kPa)	Stiffness (kPa)	CV (%)^a^	CR (kPa)^b^	ICC (95%CI)^c^
All ROIs	35.7 [29.2–43.6]	30.4 [24.8–38.9]	21.0 (17.5–24.0)	18.0 [6.2–23.6]	0.79 (0.65–0.88)
ROI 1	31.4 [26.5–41.2]	28.3 [23.1–39.4]	30.2 (23.8–35.4)	19.9 [9.6–29.3]	0.64 (0.39–0.79)
ROI 2	36.2 [27.6–47.2]	49.4 [24.7–38.4]	29.7 (23.9–34.6)	14.3 [9.9–35.3]	0.74 (0.56–0.85)
ROI 3	35.3 [29.8–49.1]	31.1 [23.9–39.9]	33.4 (26.8–38.8)	14.9 [7.7–23.7]	0.66 (0.42–0.80)
ROI 4	37.1 [29.6–45.7]	30.9 [22.3–40.6]	41.8 (35.1–47.6)	16.2 [7.8–25.2]	0.51 (0.15–0.72)

Patellar tendon stiffness was assessed using shear-wave elastography (SWE), expressed as median (interquartile range) in kPa

^a^CV: coefficient-of-variation (%), 95% confidence interval

^b^CR: coefficient-of-repeatability (kPa), also referred to as the smallest real difference (SRD)

^c^ICC: intraclass correlation coefficient (ICC), 95% confidence interval

## Discussion

In this study on the implementation of SWE on the patellar tendon in jumping athletes with patellar tendinopathy and activity-matched controls, we found that patellar tendinopathy was associated with significantly higher patellar tendon stiffness, both in univariate analyses and in adjusted multivariate analyses. The intraobserver reliability of the SWE analysis was excellent and the interobserver reliability for independent SWE acquisitions and analyses was good. This finding of tendon stiffening in PT provides additional information to GSUS/PDUS and could lead to improved understanding of the disease and eventually in altered therapeutic decision-making, for example, by staging the altered viscoelastic properties in PT and by monitoring the response to therapeutic interventions.

The trend of increased stiffness was in accordance with experiments on patellar tendon specimens that reproduced the increased state of tissue hydration in PT by using hypotonic solutions [29]. This effect may be explained by “hydraulic stiffening,” which has previously been described in bones [30]. However, the findings of SWE implementations in PT by different authors are not only different, but even contradictory: both increased [14, 31] and decreased stiffness [32, 33] in PT have been reported.

Inconsistencies in those studies included methods of image analysis, different ultrasound equipment, and different positioning of the knee. These inconsistencies form potential explanations for the discordant SWE results [15]. First, the effect of knee positioning on SWE outcome has been studied by several authors in which the same trend of increased stiffening in more flexed positions of the knee was found [34, 35]. In passive extension of the knee, we produced less physiological tensile stress on the patellar tendon which enabled to depict better contrasts in the acquired elastograms, whereas in 30 degrees of flexion, the tensile stress was much larger, which complicated the SWE acquisition. Therefore, standardized positioning of the knee is regarded as an important factor to enhance comparability of results [36]. Second, image analysis varied in other studies from a very small single ROI (1 mm diameter) in representative locations of the patellar tendon to a single ROI with flexible diameters centered in the hypoechoic region of the proximal patellar tendon [14, 33]. We evaluated average stiffness over the proximal patellar tendon as we assumed that pathological intratendinous changes are diffuse, similar to histologic findings of tissue surrounding a tendinotic lesion in Achilles tendinopathy [37, 38]. Moreover, our fixed region of interest facilitated the comparison of tendon stiffness with controls. Third, shear-wave velocities obtained with different US equipment can vary, even between different transducers and different acquisition depths [39].

Other differences of our study compared with previous studies were [1] the extensive inclusion protocol to verify the eligibility of participants, including a comprehensive physical examination with ultrasonographic confirmation as the reference standard, and [2] the assessment of intraobserver and interobserver reproducibility of SWE, which has not been reported in studies with comparable sample size. Nevertheless, the intraobserver and interobserver reliability we found were comparable with other studies using smaller sample sizes [34, 40].

Strengths of our study are the relatively large sample size and homogeneity of the study population with respect to age and level of sports. Due to our stringent inclusion criteria, the study population represented the predefined target group consisting of athletes performing sports involving frequent jumping and cutting in which PT is most prevalent. We also excluded other causes for anterior knee pain than PT such as patellofemoral pain. Patellofemoral pain is difficult to distinguish from PT without focused physical examination [20] which has led to a substantial amount of exclusions after physical examination in our study (12% of athletes who were potentially eligible after online screening). Inadequate sampling methods for athletes with anterior knee pain can potentially affect results of tendon stiffness.

The main limitation of our study is the known clinical status of the athletes before the SWE acquisition was performed, because GSUS and PDUS were part of our initial eligibility assessment in PT athletes. A second limitation is the difference in baseline characteristics between PT athletes and controls, despite the relative small differences of age and anthropometric characteristics. Therefore, we interpreted the clinical relevance of these differences as minimal. Third, the intraobserver reliability was based on analysis of multiple elastograms from one acquisition in PT athletes and interobserver reliability was based on a subset of healthy controls, where both SWE acquisitions and image analyses were performed by independent examiners.

Future research directions would comprise implementation of SWE before any reference standard is performed using standardized acquisition protocols, assessment of the prognostic value of patellar tendon stiffness in longitudinal studies, and its role to monitor therapy response.

In conclusion, SWE is able to detect higher stiffness of the proximal patellar tendon in athletes with patellar tendinopathy with a good to excellent reliability, and could provide attractive novel biomarkers to assess therapy response.

## Abbreviations

CR: Coefficient-of-repeatability
CV: Coefficient-of-variation
GSUS: Grayscale ultrasound
ICC: Intraclass correlation coefficient
kPa: Kilopascal
PDUS: Power Doppler ultrasound
PT: Patellar tendinopathy
SWE: Shear-wave elastography
VISA-P: Victorian Institute of Sports Assessment questionnaire for patellar tendons
